# (+)- Kunstlerone, a New Antioxidant Neolignan from the Leaves of *Beilschmiedia kunstleri* Gamble

**DOI:** 10.3390/molecules16086582

**Published:** 2011-08-04

**Authors:** Abbas Mollataghi, A Hamid A Hadi, Khalijah Awang, Jamaludin Mohamad, Marc Litaudon, Mat Ropi Mukhtar

**Affiliations:** 1 Department of Chemistry, Faculty of Science, University of Malaya, 50603 Kuala Lumpur, Malaysia; E-Mails: abbas@siswa.um.edu.my (A.M.); ahamid@um.edu.my (A.H.A.H.); khalijah@um.edu.my (K.A.); 2 Institute of Biological Sciences, Faculty of Science, University of Malaya, 50603 Kuala Lumpur, Malaysia; E-Mail: jamal@um.edu.my; 3 Centre de Recherche de Gif, Institut de Chimie des Substances Naturelles, CNRS, 1, Avenue de la Terrasse, 91198 Gif-sur-Yvette Cedex, France; E-Mail: marc.litaudon@icsn.cnrs-gif.fr

**Keywords:** *Beilschmiedia kunstleri*, alkaloid, neolignan, lauraceae, antioxidant

## Abstract

A new neolignan, 3,4-dimethoxy-3′,4′-methylenedioxy-2,9-epoxy-6,7-cyclo-1,8-neolign-11-en-5(5H)-one, which has been named (+)-kunstlerone (**1**), together with six known alkaloids: (+)-norboldine (**2**), (+)-*N*-methylisococlaurine (**3**), (+)-cassythicine (**4**), (+)-laurotetanine (**5**), (+)-boldine (**6**) and (**-**)-pallidine (**7**), were isolated from the leaves of *Beilschmiedia kunstleri*. The structures were established through various spectroscopic methods notably 1D- and 2D-NMR, UV, IR and LCMS-IT-TOF. (+)- Kunstlerone (**1**) showed a strong antioxidant activity, with an SC_50_ of 20.0 µg/mL.

## 1. Introduction

The Lauraceae family normally, with 30 genera and over 2,000 species, occurs throughout Southeast Asia and tropical America [1,2]. In Malaysia, its contribution is about 213 species, from 16 genera [2]. *Beilschmiedia* species are known to produce many types of phytochemicals [3,4,5,6,7,8,9,10,11,12] with varied biological activities such as *O,O*-dimethylcoclaurine isolated from Malaysian *B. brevipes,* which exhibited significant cytotoxicity against P-388 murine leukemia cells with an IC_50_ value of 6.5 μg/mL [13]. Besides the alkaloids, epoxyfuranoid lignans were also reported in the leaves of *B. tsangii* [10]. Neolignans, whose precursors are the di- and trioxygenated cinnamic acids, have never been reported so far in the species of *Beilschmiedia;* however they were detected in parts of other species of Lauraceae such as in the fruits of *Aniba riparia* and in the leaves of *Ocotea catharinensis* [14,15,16]. 

**Figure 1:**
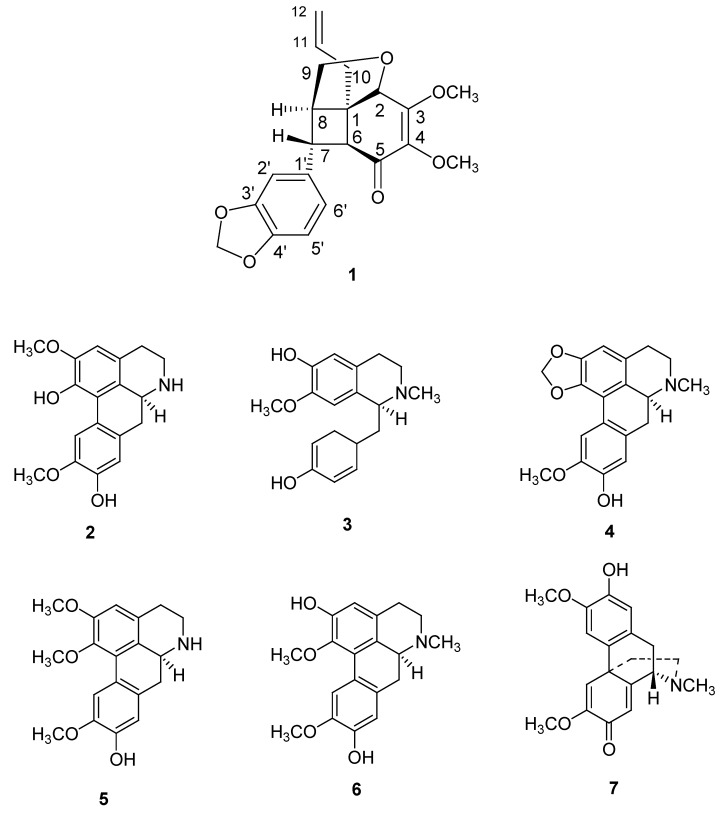
Chemical structures of compounds **1-7**.

In continuation of our search for new bioactive compounds from Malaysian flora [13,17], we have performed a phytochemical study on the leaves of a Malaysian Lauraceae, *Beilschmiedia kunstleri*, which has led to the isolation of a new neolignan; (+)-kunstlerone (**1**). In addition, six known isoquinoline alkaloids, namely (+)-norboldine (**2**) [18], (+)-*N*-methylisococlaurine (**3**) [19], (+)-cassythicine (**4**) [20], (+)-laurotetanine (**5**) [21], (+)- boldine (**6**) [17] and (-)-pallidine (**7**) [22] were also isolated (Figure 1). This paper describes the structural elucidation and the DPPH radical scavenging activity of (+)-kunstlerone (**1**). 

## 2. Results and Discussion

(+)-Kunstlerone, $\left[\propto \right]{\;}_{D}^{25}$ = +163.63° was obtained as a white amorphous solid. The LCMS-IT-TOF revealed a pseudomolecular ion peak at *m/z* 371.1490 [M+H]^+^, thus suggesting a molecular formula of C_21_H_23_O_6_ (calc. 371.1495). The IR spectrum revealed absorption bands at 1,615 and 1,725 cm^−1^ due to the C=C and C=O stretching vibrations, respectively [14]. The ^1^H-NMR spectrum (see Table 1) established the presence of three ABX type phenyl protons at δ 6.76 (1H, *d*, *J* = 8.1 Hz, H-5′), 6.71 (1H, *br d*, *J =* 8.1 Hz, H-6′), and 6.80 (1H, *br s*, H-2′)], two methoxyl groups at δ 4.08 and 3.69, a methylenedioxy (δ 5.91, *s*, 2H), and protons of a propenyl group; H-11( δ 5.66, m), H*_tran_*-12 **(**δ 5.11, *br d*, 16.5 Hz) and H*_cis_* -12 (δ 5.13, *br d*,11.0 Hz), respectively. The proton at C-7 of a cyclobutane moiety showed significantly as a *dd* at δ 3.21 with coupling constants of 7.8 and 8.5 Hz, which indicated that it is *trans* to H-8 (δ 2.83 *m*) and H-6 (δ 2.90 *br d*, 8.5 Hz) [23,24]. 

**Figure 2:**
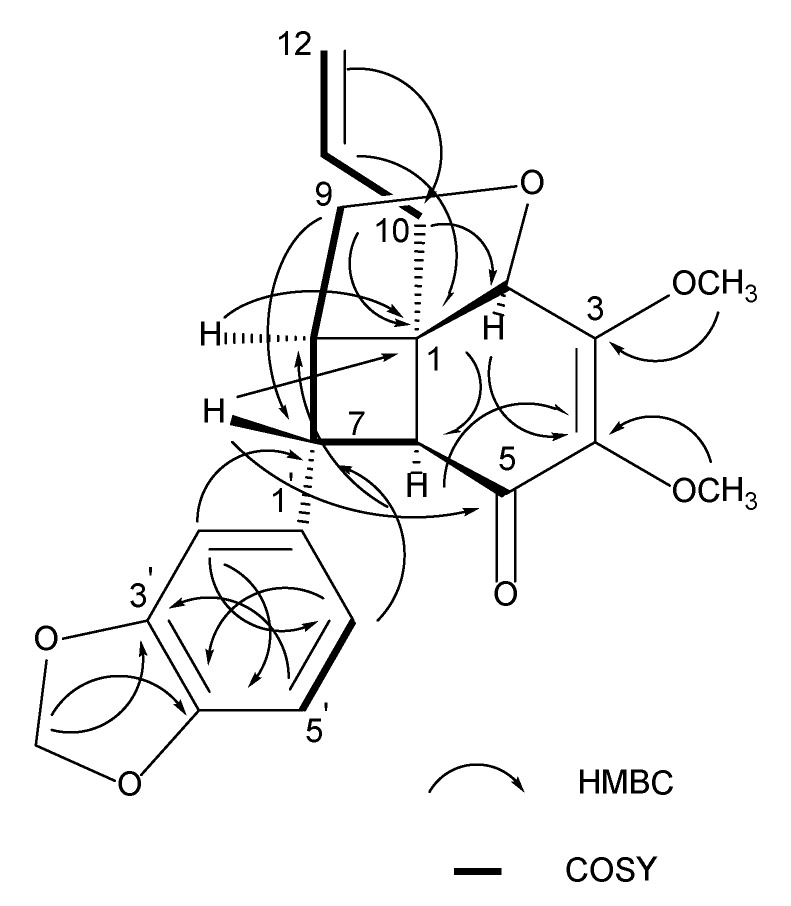
Selected HMBC and COSY correlations of (+)-kunstlerone (**1**).

The ^13^C-NMR spectrum (Table 1) indicated the presence of 21 carbon signals; two methyls, four methylenes, four sp^2^ methines, four sp^3^ methines, six quaternary carbons and one carbonyl. The sp^3^ quaternary carbon, C-1 bearing a propenyl group gave a signal at δ 46.0, while C-5 carbonyl peak was observed at δ 193.1. The correlations of H-7/C-1, H-9/C-1 and H-11/C-1 in the HMBC spectrum further confirmed the position of propenyl fragment at C-1. In addition, the cross-peaks in the COSY spectrum were observed between H-5′/H-6′, H-7/H-6, H-7/H-8, H-8/H-9, H-10/H-11and H-11/H-12 as shown in Figure 2. Finally, thorough analysis of the COSY, HMQC and HMBC spectra allowed the complete assignments of all protons and carbons of compound **1** (Table 1). 

**Figure 3:**
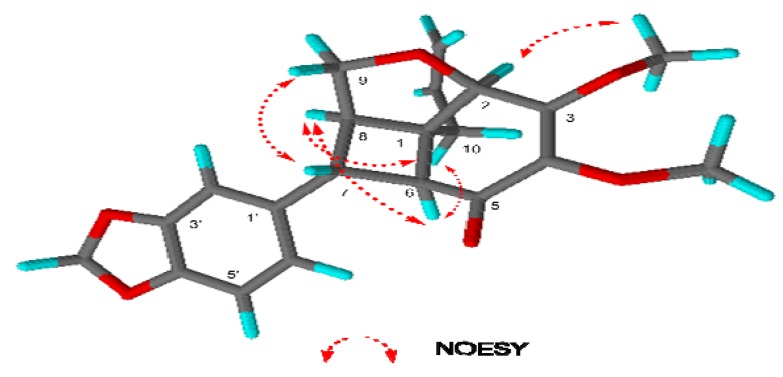
Selected NOESY correlation of (+)- kunstlerone (**1**).

The relative configuration of the five stereogenic centers of compound **1** was determined by the correlations between H-6 (δ 2.90)/H-8 (δ 2.83), H-2/3-OCH_3_ (δ 4.08), H-6/H-10(δ 2.38) and H-7/H-9(δ 3.90) in the NOESY spectrum. Therefore, the relative configurations were assigned as 7*R*, 8*R*, 1*R*, 6*S* and 2*R*, as depicted in Figure 3 [24].

### Antioxidant activity

**Figure 4:**
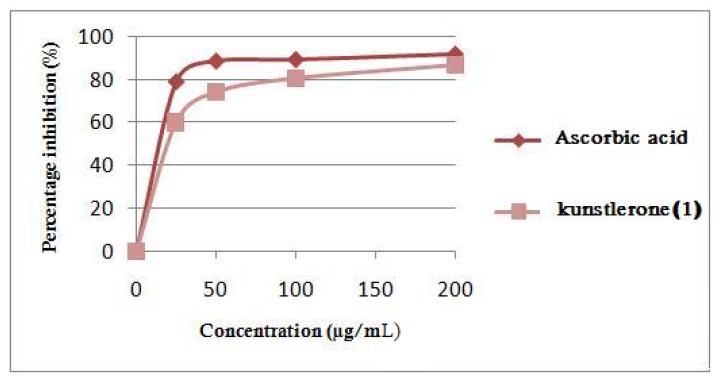
DPPH radical scavenging activity of the isolated new neolignan; (+)- kunstlerone (**1**) with an SC_50_ = 20.0 µg/mL.

(+)- Kunstlerone (**1**) was tested for antioxidant activity using a DPPH radical scavenging activity assay. The results have shown that it possesses a potent antioxidant activity, with scavenging capacity (SC_50_ = 20.0 µg/mL) comparable to that of ascorbic acid (SC_50_ = 14.0 µg/mL, Figure 4). The antioxidant activities of neolignan glycosides from *Verbascum salviifolium* Boiss and neolignan phenols from *Nectandra grandiflora* have been reported previously [25]. The mechanism of action for the strong scavenging capacity of compound **1** is under investigation.

## 3. Experimental

### 3.1. General

The optical rotations were recorded on a JASCO (Japan) P1020 Polarimeter equipped with a tungsten lamp; MeOH as solvent. The mass spectra were obtained from LCMS-IT-TOF, Shimadzu. The ultraviolet spectra were obtained in MeOH on a Shimadzu UV-310 ultraviolet-visible spectrometer. The Fourier Transform Infrared (FTIR) spectra were obtained with CHCl_3_ (NaCl window technique) on a Perkin Elmer 2000 instrument. The ^1^H-NMR and ^13^C-NMR spectra were recorded in deuterated chloroform on a JEOL 400 MHz spectrometer; chemical shifts are reported in ppm on δ scale, and the coupling constants are given in Hz. Mayer’s reagent was used for alkaloid screening. Aluminum TLC sheets and PTLC (20 × 20 cm Silica gel 60 F_254_) were used in the TLC analysis. The TLC and PTLC spots were visualized under UV light (254 and 366 nm) followed by spraying with Dragendorff’s reagent for an alkaloid detection. All solvents, except those used for bulk extraction, were AR grade.

### 3.2. Plant materials

The leaves of *Beilschmiedia kunstleri* (Lauraceae) was collected from Hutan Simpan Sungai Tekam, Jerantut, Pahang, Malaysia. The plant was identified by Mr. Teo Leong Eng. A voucher specimen (KL5627) was deposited at the Herbarium of the Department of Chemistry, University of Malaya, Kuala Lumpur, Malaysia and at the Herbarium of the Forest Research Institute, Kepong, Malaysia.

### 3.3. Extraction and isolation of neolignan and the alkaloids

The air-dried leaves (2.50 kg) of *Beilschmiedia kunstleri* Gamble were extracted exhaustively with hexane (10.0 L) for 72 hours. The residual plant material was dried and left for 4 h after moistening with 10% NH_4_OH. It was then macerated with CH_2_Cl_2_ (12.0 L) for four days. After filtration, the supernatant was concentrated to 500 mL at room temperature (30 °C) followed by acidic extraction with 5% HCl until a negative Mayer’s test result was obtained. The aqueous solution was made alkaline to pH 11 with NH_4_OH and re-extracted with CH_2_Cl_2_. This was followed by washing with distilled H_2_O, dried over anhydrous sodium sulphate, and evaporation to give a crude alkaloid (4.50 g). The crude alkaloid (4.00 g) was subjected to exhaustive column chromatography over silica gel (column diameter = 2 cm, length =75 cm, silica gel 60, 70–230 mesh ASTM; Merck 7734) using CH_2_Cl_2_ gradually enriched with methanol (1% → 80% MeOH; 450 mL portions of eluent were used for each percentage) to yield 12 fractions. Purification of combined fractions 3–5 (0.4 g) using preparative TLC (Merck KGaA silica gel 60 F_254_) afforded a neolignan identified as kunstlerone (**1**, 0.02%), pallidine (**7**, 0.08%, CH_2_Cl_2_-MeOH; 98:2) and norboldine (**2**, 0.07%, CH_2_Cl_2_-MeOH; 95:5). Fraction 9 afforded *N*-methylisococlaurine (**3**, 0.05%, CH_2_Cl_2_-MeOH; 98:2), and fractions 10–11 (0.3 g) produced cassythicine (**4**, 0.03%; CH_2_Cl_2_-MeOH; 97:3), laurotetanine (**5**, 0.01%, CH_2_Cl_2_-MeOH; 98:2) and boldine (**6**, 0.04%, CH_2_Cl_2_-MeOH; 98:2).

*(+)-Kunstlerone* (**1**, Figure 1).$\;\left[\propto \right]{\;}_{D}^{25}$ = +163.63° (C = 2.37 × 10^−3^ M, MeOH), was obtained as a white amorphous solid; UV max (MeOH): 404 (1.36) and 332 (4.00) nm; IR bands (KBr): 1,725, 1,654, 1,615, 1,491, 1,231, and 1,038 cm^−1^; ^1^H-NMR (400 MHz, CDCl_3_) and ^13^C-NMR (100 MHz, CDCl_3_): see Table 1; LCMS-IT-TOF, m/z*:* 371.1490 [M+H]*^+^* (calc. 371.1495 for C_21_H_23_O_6_). 

### 3.4. Antioxidant assay

The free radical scavenging activity was determined using DPPH as described by Shimada *et al.* [26]. The DPPH radical scavenging activity assay is a decolorization assay that determines the activity of antioxidants to directly react with DPPH stable free radical by observing its absorbance at 517 nm with a spectrophotometer. A purple colored 1,1-diphenyl-2-picryl hydrazyl (DPPH), a stable free radical which is reduced to *α,α*-diphenyl-*β*-picryl hydrazine and give yellow color when reacts with antioxidant. The decolorization of purple color indicates the potential of antioxidants of the samples in which increased decolorization shows the higher scavenging activity of the samples. 

Briefly, 0.1 mM DPPH (1 mL) dissolved in ethanol was added to an ethanol solution (3 mL) of the tested compound at different concentrations (25, 50, 100, 150, 200 µg/mL). An equal volume of ethanol was added in the control test. The mixture was shaken vigorously and allowed to stand at room temperature for 30 min. Then the absorbance at 517 nm was measured with a UV–VIS spectrophotometer. Lower absorbance of the reaction mixture indicated higher free radical scavenging activity. The percentage of scavenging of DPPH was calculated using the following equation:

$$\mathrm{DPPH}\;\mathrm{scavenging}\;\mathrm{effect}\;(\%)=\frac{A{}^{\circ}-A1}{A{}^{\circ}}\times 100$$

where A^°^ is the absorbance of the control reaction and A1 is the absorbance in the presence of the sample.

## 4. Conclusions

**Scheme 1:**
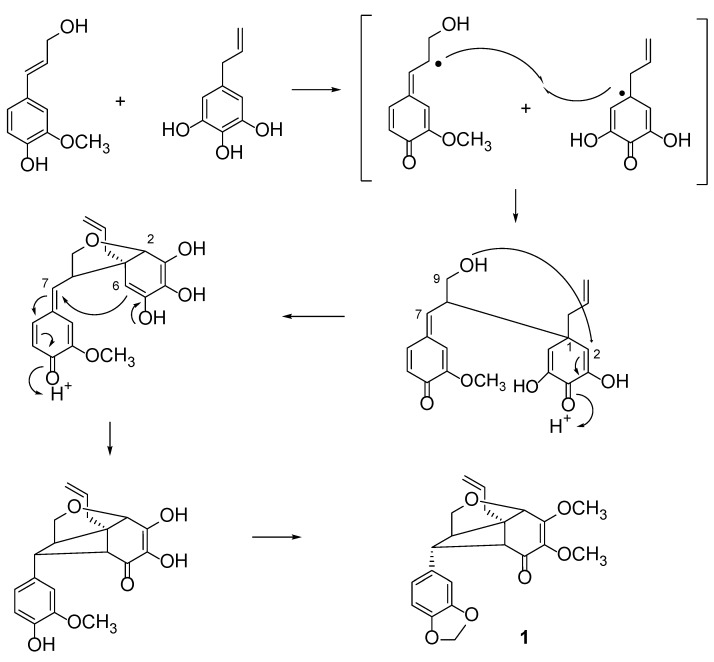
Biogenetic pathway for kunstlerone (**1**).

This is the first communication on neolignans from the *Beilschmiedia Kunstleri*. To the knowledge of the authors, kunstlerone (**1**) is the first neolignan reported in the family of Lauraceae bearing a propenyl group at C-1 and the ether ring that is attached to C-2 and C-9. (+)-Kunstlerone (**1**) showed a strong antioxidant activity with an SC_50_ of 20.0 µg/mL. This compound was also detected in neutral fractions, showing that it could be isolated using an acid and base extraction. A proposed plausible biogenetic pathway for **1** as shown in Scheme 1. Apparently, it results from the oxidative coupling between the two precursors coniferyl alcohol and allyl-3,4,5-trihydroxybenzene. The primary hydroxyl would give a Michael-type addition to *α*,*β*-carbonyl function and then, an enol addition to the quinone methide (attack of C-6 to C-7) would give the cyclobutane ring. Finally, the dimer was *O*-methylated at C-3 and C-4 and the methylenedioxy group was formed between the hydroxyl and methoxyl groups of the coniferyl alcohol moiety. Alkaloids **2**-**7**, which were identified in this study, belong to the aporphine, benzylisoquinoline and morphinandienone type of alkaloids. 

## Figures and Tables

**Table 1:** ^1^H-NMR and ^13^C-NMR spectral data of (+)-kunstlerone (**1**) in CDCl_3_ (δ in ppm, *J* in Hz).

Position	^1^H	^13^C
C-1′	^-^	132.12
C-2′	6.80, br s	107.25
C-3′	^-^	147.97
C-4′	^-^	146.39
C-5′	6.76, d, (8.1)	108.32
C-6′	6.71, br d, (8.1)	119.69
C-1	^-^	46.01
C-2	4.32, s	79.11
C-3	^-^	159.47
C-4	^-^	138.46
C-5	^-^	193.10
C-6	2.90, d, (8.5)	50.16
C-7	3.21, dd, (7.8, 8.5)	44.58
C-8	2.83, m	49.63
C-9	3.72, dd, (9.5, 3.9) 3.90, d, (9.5)	70.84
C-10	2.38, m	41.76
C-11	5.66, m	136.51
C-12	5.13, d, (*J*_cis_ = 11.0), 5.11, d, (*J*_trans_ = 16.5)	119.41
OCH_2_O	5.91, s	101.08
3-OCH_3_	4.08, s	58.46
4-OCH_3_	3.69, s	60.57
